# Long Noncoding RNAs as Diagnostic and Therapeutic Targets in Type 2 Diabetes and Related Complications

**DOI:** 10.3390/genes8080207

**Published:** 2017-08-22

**Authors:** Fatjon Leti, Johanna K. DiStefano

**Affiliations:** Department of Biomedical Research, National Jewish Health, Denver, CO 80210, USA; letif@njhealth.org

**Keywords:** diabetes, diabetic kidney disease, diabetic retinopathy, microvascular complications, noncoding RNA, lncRNA, epigenetics, siRNA, antisense oligonucleotides

## Abstract

Protein-coding genes represent only a small fraction of the human genome. In the past, the majority of the genomic sequence has been considered transcriptionally silent, but recent large-scale studies have uncovered an array of functionally significant elements, including non-protein-coding transcripts, within these noncoding regions of the human genome. Long noncoding RNAs (lncRNAs), a class of noncoding transcripts with lengths >200 nucleotides, are pervasively transcribed in the genome and function as signals, decoys, guides, or scaffolds to regulate gene expression. More than 200 diseases have been associated with dysregulated or dysfunctional lncRNAs, and new associations continue to accumulate in the literature. The role of lncRNAs in the pathogenesis of type 2 diabetes mellitus and related complications has only recently been recognized, but there is already evidence for their involvement in many of the pathophysiological mechanisms underlying the disease. In this review, we summarize the current knowledge of the functions and underlying mechanisms of lncRNA activity with a focus on type 2 diabetes mellitus and related renal and retinal complications of the disease. We also discuss the potential of lncRNAs to serve as therapeutic targets for drug development and diagnostic markers for clinical applications in the management of diabetes.

## 1. Introduction

### 1.1. Long Noncoding RNAs are Noncoding, Multifunctional Transcripts

Although a large majority of the human genome is transcribed, only a small proportion of transcribed sequence gives rise to protein products [1,2]. The remainder of the transcribed human genome is comprised mostly of noncoding RNAs (ncRNA), which are broadly represented by infrastructural and regulatory transcripts. Most infrastructural ncRNAs are constitutively expressed and have recognized roles in splicing and translation [3]. However, some infrastructural ncRNAs perform regulatory functions, contribute to chromosome maintenance and segregation, and participate in protein targeting, suggesting a greater diversity of functionality than previously appreciated [3]. Regulatory ncRNAs constitute a wide array of molecules, including, for example, microRNAs (miRNAs), enhancer RNAs (eRNAs), and piwi-interacting RNAs (pi-RNAs), all of which function to modulate gene expression [3,4]. Over the past several years, discovery of new ncRNA species and functions has highlighted the complexity with which these molecules contribute to the regulation of gene expression and cell biology.

Long noncoding RNAs (lncRNAs) belong to a heterogeneous class of regulatory ncRNAs with transcript lengths >200 nucleotides [5]. Like mRNAs, most lncRNAs are transcribed by RNA polymerase II, utilize common consensus splicing signals, and are oftentimes polyadenylated [5,6,7]. A number of functions have been ascribed to lncRNAs, most of which involve roles as signals, decoys, guides, or scaffolds [8,9]. As regulators of gene expression, lncRNAs can exert *cis*- or *trans*-acting effects [10]. *Cis*-acting lncRNAs silence or activate expression of genes located on the same chromosome, while *trans*-acting lncRNAs affect genes on chromosomes other than the one from which they are transcribed and regulate gene expression through recruitment of proteins to target sites or sequestration of transcription factors away from targeted sites of transcription [11].

Most lncRNAs are characterized by localization to the nucleus, reduced conservation across species, shorter sequence lengths, and fewer exons compared with protein-coding transcripts [6,12,13]. In addition, lncRNAs show stronger tissue-specific patterns of expression relative to mRNAs [14]. Up until recently, lncRNAs were believed to have low levels of expression [15]. However, single-cell sequencing studies have revealed abundant lncRNA expression in individual cells [16,17], suggesting that cell-specific enrichments may have been masked in expression analyses using RNA extracted from whole tissue. Abundant cell-specific lncRNA expression may prove useful in the development of targeted therapeutics, where expected benefits could be directed to the cell of interest without incurring undesirable effects in off-target cell types.

A growing body of literature is revealing a role for lncRNAs in the development and progression of a number of different diseases. Dysregulation or functional alteration of lncRNAs has been implicated in the pathogenesis of many kinds of cancer [18,19,20,21,22], Alzheimer’s disease [23,24,25], human immunodeficiency virus (HIV) infection [26,27], and other diseases [28,29,30,31]. More than 200 diseases have been associated with dysregulated or dysfunctional lncRNAs, and new associations continue to accumulate in the literature [32]. In parallel with their role in disease pathogenesis, lncRNAs may also serve as markers of disease status and aid in the diagnosis, prognosis, and clinical management of disease, particularly cancer [33,34]. For example, peripheral blood [21] and plasma [35] levels of the lncRNA HULC (highly upregulated in liver cancer) are substantially higher in patients with hepatocellular carcinoma compared to individuals with no evidence of liver disease, and reflect expression patterns in the liver [21]. Corroboration between circulating and neoplasmic expression patterns thus provides a foundation upon which HULC might be developed as a noninvasive biomarker for the diagnosis and prognosis of hepatocellular carcinoma. The diagnostic potential of lncRNAs is discussed in greater detail below.

### 1.2. Diabetes Is a Worldwide Health Concern

Diabetes mellitus is a heterogeneous collection of disorders resulting from a deficiency or failure to maintain normal glucose homeostasis. Type 2 diabetes (T2D) develops in response to multi-organ insulin resistance and inadequate insulin secretion from pancreatic β-cells [36,37]. Due to a combination of different factors, including increased levels of sedentary behavior and easy access to high-energy foods, the prevalence of T2D has risen in recent decades and today affects more than 300 million individuals worldwide [38]. Current estimates project that T2D will affect more than 520 million individuals within 15 years [39]. At the time of this writing, T2D is the eighth leading cause of death worldwide and accounts for approximately 2.7% of all deaths [40]. For most industrialized countries, T2D represents a major public health concern.

The pathogenesis of T2D is complex and multifactorial, and genetic predisposition, environmental exposures, and lifestyle factors modulate susceptibility to the disease [41]. Despite intense research efforts to identify biological targets and signaling pathways related to these factors, the molecular mechanisms by which environmental influences affect the pathogenesis of T2D in susceptible individuals remain largely unknown. Furthermore, while obesity, increased sedentary behavior, and aging are known to increase the risk of developing T2D, there is also a substantial proportion of people who do not develop the disease in the face of these exposures. Genome-wide association studies have identified a number of polymorphisms associated with T2D and related complications, although together these account for only a small proportion of the inter-individual variation in disease susceptibility [42,43,44,45]. Nearly 90% of the variants identified to date map to non-protein coding regions, a finding that challenges our understanding of the functional impact of such genetic factors [1,2,46]. Growing evidence implicates ncRNAs in the etiology of T2D and given the expression patterns and function of lncRNAs in specific, these molecules may represent potential mediators of environmental influences on pathological mechanisms underpinning T2D and microvascular complications of the disease [47].

### 1.3. LncRNA Profiling in Pancreatic β-Cells and Regulation of Glucose Homeostasis

The role of ncRNAs in the pathogenesis of T2D has only recently become recognized, yet a growing list of lncRNAs involved in glucose homeostasis is emerging [48]. Here, we present findings from lncRNA profiling studies in pancreatic β-cells and islets and discuss specific candidates that have been experimentally characterized under the auspices of focused investigations (Table 1).

Pancreatic β cells produce, store, and release insulin, and play a critical role in the maintenance of glucose homeostasis [72]. The deterioration or loss of these cells leads to an inability to meet the increasing demands for insulin needed by the body to maintain glucose homeostasis, eventually resulting in the development of diabetes mellitus. As discussed in the following sections, lncRNAs contribute to biological processes relevant to the control of glucose homeostasis; however, recent work has also shown a role for these molecules in pancreatic development. For example, homozygous deletion of the β-cell long intergenic noncoding RNA (*βlinc1*) in mice resulted in the downregulation of critical islet-specific transcription factors and impaired the specification and function of β-cells in embryonic development [51]. Not surprisingly, deletion of *βlinc1* in adult mice corresponded with a reduction in the total number of β-cells and led to glucose intolerance in these animals.

Recent profiling studies have uncovered thousands of lncRNAs in human pancreatic β cells. In a study of human pancreatic islet and β-cells, over 1000 lncRNAs were found to be islet-specific and >19% of the islet transcriptome mapped outside of annotated protein-coding genes [73]. The majority of lncRNAs identified were either silent or expressed at very low levels in pancreatic progenitors, but were active in adult islets, indicating potential roles in the regulation of pancreatic endocrine differentiation. In an investigation of 55 T2D susceptibility loci, nine contained islet lncRNAs within 150 kb of the reported lead marker, including six that have been linked directly to β-cell dysfunction [74,75,76,77,78]. ANRIL (also known as CDKN2B antisense RNA 1) has been found to harbor genetic variants associated with T2D [79]. ANRIL maps to the *INK4* locus and is required for the silencing of the p15^INK4B^ tumor suppressor gene [49]. Variants in ANRIL that disrupt its expression or function may affect compensatory increases in pancreatic β-cell mass in response to increasing demands for insulin in the pre-diabetes state [80].

In pancreatic islets from 89 donors with varying degrees of glucose tolerance, nearly 500 islet long intergenic noncoding RNAs (lincRNAs) were identified, 54 of which were associated with gene expression and exon use [58]. Seventeen lincRNAs were associated with HbA1c levels, including HI-LNC901 (i.e., LOC283177), whose expression was associated with a genetic variant (rs73036390) and also directly correlated with insulin exocytosis. HI-LNC901 was co-expressed with mitogen-activated protein kinase activating death domain (MADD), synaptotagmin 11 (SYT11), and paired box 6 (PAX6), all of which have been implicated in islet function.

A recent study applied transcript knockdown and co-expression network analysis to investigate the function of β-cell-specific lncRNAs and transcription factors, focusing on a set of 12 lncRNAs showing restricted β-cell expression and lying in proximity to protein-coding genes with important functions in β-cells [70]. The analysis revealed a network of lncRNAs and transcription factors that regulate β-cell-specific transcriptional networks. lncRNAs were also found to regulate genes associated with clusters of islet enhancers, the predominant functional target of islet-specific transcription factors. Characterization of PLUTO (also known as HI-LNC71), an lncRNA located upstream of pancreatic and duodenal homeobox 1 (*PDX1*), a transcription factor involved in pancreas development and β-cell function, revealed that the lncRNA affected contacts between the *PDX1* promoter and distal enhancers, causing a decrease in *PDX1* transcriptional activity. Both *PLUTO* and *PDX1* were downregulated in islets from donors with T2D or impaired glucose tolerance. Together, these data are among the first to implicate cell-specific lncRNAs in human β-cell transcriptional programs and suggest potential applications for the manipulation of β-cell differentiation, function, or mass in the treatment and prevention of diabetes.

In addition to profiling investigations in β-cells and pancreatic islets, a number of studies have focused on analysis of individual lncRNAs. One of these lncRNAs, H19, encodes a maternally expressed transcript [81] and plays a role in cell proliferation [56], regulation of gene expression, and development of cancer [57]. H19 is located on chromosome 11p15.5, approximately 100 kb distal of insulin-like growth factor 2 (*IGF2*), and together H19 and IGF2 are transcribed from a conserved imprinted gene cluster [56]. In female mice carrying pups with targeted disruption of H19, final trimester glucose concentrations were significantly higher compared to wild-type animals [82]. In humans, maternally transmitted H19 alleles were associated with increased birth weight, increased head circumference, and increased sum of skinfold thicknesses in offspring [83]. In T2D patients, H19 levels in skeletal muscle were approximately five times lower than those in healthy individuals [84].

Under normal conditions, H19 sequesters lethal 7 (let-7) miRNA, preventing interactions with key target genes such as insulin receptor (*INSR*) and lipoprotein lipase (*LPL*) [85]. In diabetes, where H19 expression is decreased, let-7 levels increase, leading to greater inhibition of *INSR* and *LPL* expression and resulting in dysregulated glucose metabolism in skeletal muscle. H19 expression is also downregulated by hyperinsulinemia through a pathway involving phosphatidylinositide 3-kinase/protein kinase B (PI3K/AK)-dependent phosphorylation of the miRNA-processing factor KH-type regulatory splicing protein (KSRP), which promotes let-7 biogenesis and subsequent H19 destabilization. Thus, a double-negative feedback loop between H1*9* and let-7 may function to regulate glucose homeostasis in skeletal muscle.

*MEG3* (maternally expressed 3) gene has also been explored as a candidate lncRNA in the regulation of glucose homeostasis. Like H19, MEG3 is a maternally expressed imprinted lncRNA [86], with established roles in cell proliferation [87,88,89]. In the obese (ob/ob) mouse model of T2D and high-fat diet-fed mice [65], MEG3 levels were elevated relative to control animals. In primary hepatocytes, MEG3 overexpression was associated with increased hepatic gluconeogenesis and suppressed insulin-stimulated glycogen synthesis. In response to MEG3 overexpression, levels of forkhead box O1 (FOXO1), glucose-6-phosphatase catalytic subunit (G6PC), and phosphoenolpyruvate carboxykinase (PEPCK) increased, while levels of palmitate-induced FOXO1, G6PC, and PEPCK were reversed with MEG3 downregulation. MEG3 knockdown in high-fat diet-fed or ob/ob mice reversed triglyceride upregulation, impaired glucose tolerance, and downregulation of glycogen content.

In addition to hepatic effects, MEG3 affects insulin synthesis and secretion in pancreatic β-cells [66]. While MEG3 expression was higher in islets compared to exocrine glands in BALB/c mice, islet expression of MEG3 was reduced in non-obese diabetic (NOD) mice, a model of type 1 diabetes, and db/db mice, a model of T2D. Glucose was found to regulate MEG3 expression in isolated mouse islets and a pancreatic β-cell line [66]. Downregulation of MEG3 expression in vitro corresponded with decreased insulin synthesis and secretion and increased β-cell apoptosis, consistent with in vivo findings of impaired glucose tolerance and reduced insulin secretion. MEG3 knockdown also corresponded with decreased levels of two transcription factors: PDX1 (discussed earlier) and v-maf musculoaponeurotic fibrosarcoma oncogene family, protein A (MAFA), both of which are critical for β-cell-specific regulation of insulin gene expression.

In another study, genome-wide lncRNA profiling in macrophages isolated from diabetic db/db and db/+ control mice revealed more than 170 differentially expressed lncRNAs between the two groups [53]. Among these differentially expressed lncRNAs, E330013P06 (E33) showed high expression and strong upregulation lncRNA in diabetic mice compared to the control animals. Levels of E33 were increased in macrophages from db/db mice and diet-induced insulin resistant T2D mice, but not streptozotocin (STZ)-induced diabetic mice (a model of Type 1 diabetes, T1D), indicating that insulin resistance and T2D selectively contribute to changes in *E33* levels. In addition to E33 upregulation in T2D, treatments with high glucose and palmitic acid increased its expression in macrophages from db/+ mice, which corresponded with elevated levels of inflammatory genes. E33 knockdown in macrophages from db/db mice did not affect basal levels of inflammatory genes, but significantly attenuated its expression in response to treatments with high glucose and palmitic acid. These results suggest that E33 contributes to a proinflammatory phenotype in diabetic macrophages, and present evidence that lncRNAs have the potential to serve as therapeutic targets to reduce inflammation in T2D.

Expression levels of the human homologue of E33, MIR143HG (RefSeq NR_105060.1) were also increased in monocytes derived from T2D patients. Both E33 and MIR143HG host miR-145 and miR-143, the latter of which is associated with insulin resistance [90]. Levels of miR-143, but not miR-145, were increased in macrophages of db/db mice compared to db/+ animals, suggesting that this E33-derived miRNA may be regulated post-transcriptionally at the level of biogenesis [53].

Although relationships between lncRNAs and miRNAs are well established, emerging studies indicate the presence of even more complex regulatory networks among coding transcripts, lncRNAs, and miRNAs in the pathogenesis of cancer, muscular dystrophy, and neurodegenerative diseases [91,92,93]. Lin et al. [68] constructed a T2D-related network of 98 mRNAs, 86 miRNAs, and 167 lncRNAs to explore the biological functions of lncRNAs during the development of diabetes. Focusing on the mechanistic target of rapamycin (mTOR) network because of its recognized role in energy metabolism, one gene (*mTOR*), three miRNAs, and 15 lncRNAs were identified and interactions between miR-181b-MALAT1, miR-144-MALAT1, and miR-181b-NEAT1 were detected. MTOR-associated protein, LST8 homolog (mLST8) was identified as a potential miR-181b target, and treatment with miR-181b mimics or nuclear paraspeckle assembly transcript 1 (NEAT1) silencing RNA (siRNA) reduced mLST8 protein levels and mLST8 luciferase activity, demonstrating that NEAT1-miR-181b-mLST8 interaction regulates the mTOR signaling pathway. These results are among the first to provide evidence supporting a lncRNA regulatory network in T2D. Additional studies are necessary to further explore the concept of RNA networks in the dysregulation of glucose homeostasis.

### 1.4. LncRNA Profiling in Diabetic Kidney Disease

Diabetic kidney disease is a progressive microvascular complication of diabetes caused by damage to the capillaries of the glomeruli and characterized by persistent albuminuria, declining glomerular filtration rate (GFR), and elevated arterial blood pressure [94]. Other components of diabetic kidney disease include glomerulopathy, mesangial cell proliferation, and extracellular matrix (ECM) accumulation [95,96], as well as impaired autophagy and increased intra-renal nitric oxide (NO) production [97,98]. Diabetic kidney disease contributes to most of the reduced life expectancy in patients with diabetes [99,100,101,102]. The annual incidence of diabetic kidney disease has more than doubled in the past decade [103], and today accounts for almost 50% of all end-stage renal disease [96].

Although the investigation of lncRNAs in diabetic kidney disease is relatively new, there are already several profiling and candidate gene-focused studies supporting the involvement of these molecules in disease pathogenesis and progression. Here, we provide a discussion of major findings from profiling experiments and studies of individual lncRNAs that have emerged in recent years (Table 1).

In a microarray-based study comparing renal lncRNA expression patterns between a mouse model of diabetic nephropathy and a db/m control, 311 differentially expressed transcripts were detected [104]. Gene ontology enrichment and Kyoto Encyclopedia of Genes and Genomes (KEGG) analysis of protein-coding genes co-expressed with these lncRNAs identified pathways targeted to the Golgi apparatus, catalytic activity, and mitotic nuclear division, and were mostly enriched in glutathione metabolism signaling. Nearly half of the dysregulated lncRNAs were *cis*-regulatory, suggesting that these transcripts function, in part, to regulate protein-coding genes in biological pathways related to the pathogenesis of diabetic kidney disease.

In another profiling study, microarray analyses performed using renal cortical tissues dissected from kidneys of db/db and db/m mice identified several differentially expressed lncRNAs and nearby mRNAs [54]. One candidate, ENSMUST00000147869, which was proximally associated with Cyp4a12a (cytochrome P450, family 4, subfamily a, polypeptide 12a), was selected for functional characterization. In mouse mesangial cells, expression of both ENSMUST00000147869 and Cyp4a12a was downregulated in response to glucose. Overexpression of ENSMUST00000147869 in mesangial cells not only correlated with increased Cyp4a12a expression, but was also associated with decreased levels of proliferating cell nuclear antigen (PCNA), cyclin D1, fibronectin, and collagen 1, suggesting a role for this lncRNA in processes related to proliferation and fibrosis in the mesangium.

Inflammation plays a role in the development of diabetic kidney disease and activation of nuclear factor kappa light-chain enhancer of activated B cells (NF-κB) is associated with renal inflammation and fibrosis in the progression of the disease [55]. Profiling studies of renal lncRNAs in db/db mice and mesangial cells cultured under high glucose conditions identified 12 transcripts displaying the same expression patterns in the two datasets [105]. The most upregulated lncRNA, Gm4419 (ENSMUST00000180671), was found to regulate levels of pro-inflammatory cytokines (chemokine (C-C motif) ligand 2, tumor necrosis factor-α, and interleukin 1 β) and extracellular matrix components (fibronectin and collagen IV) in mesangial cells cultured under high (25 mM) or low (5.5 mM) glucose conditions. Overexpression of GM4419 corresponded with increased levels of p50 and p65, key molecules in the NF-κB pathway in the presence of low glucose concentrations, while knockdown of the lncRNA resulted in a decrease of p50 and p65 under high glucose conditions. Gm4419 was found to activate the NF-κB pathway through direct interaction with p50, the subunit of NF-κB. In addition, p50 was found to interact with the NACHT, LRR and PYD domains-containing protein 3 (NLRP3) inflammasome in mouse mesangial cells. Together, these findings indicate that Gm4419 may participate in the inflammation, fibrosis and proliferation in mesangial cells under high-glucose conditions through the NF-κB/NLRP3 inflammasome signaling pathway. Further investigation of this network in other models is necessary before these results can be extrapolated to disease pathogenesis in humans.

In addition to lncRNA profiling investigations, a number of studies have analyzed individual transcripts within the context of biological processes related to renal dysfunction in T2D. Here we briefly summarize several lncRNA candidates that have been implicated in the development of diabetic kidney disease.

Expression of cytochrome P450, family 4, subfamily b, polypeptide 1, pseudogene 1 (CYP4B1-PS1-001) was downregulated in kidney tissue from obese db/db mice with early symptoms of diabetic nephropathy, compared to control db/m animals [52]. Under hyperglycemic conditions, CYP4B1-PS1-001 overexpression reversed proliferation of mouse mesangial cells and was associated with decreased levels of PCNA, cyclin D1, fibronectin, and collagen I. Under low glucose conditions, CYP4B1-PS1-001 knockdown corresponded with increased expression of PCNA, cyclin D1, and fibronectin in mouse mesangial cells, providing evidence that this lncRNA may regulate proliferation and fibrosis in mesangial cells. The role of CYP4B1-PS1-001 in other renal cells and human mesangial cells warrants further investigation.

Sequencing of RNA extracted from glomeruli of STZ-induced diabetic mice revealed significant upregulation of a cluster of miRNAs within the DLK-DIO3 genomic region compared to untreated control animals [59]. This cluster of approximately 40 miRNAs is contained within a single lncRNA, named lnc-MGC (lncRNA-megacluster). Glomerular expression of both the miRNA megacluster and lnc-MGC was increased in STZ-treated mice and mesangial cells treated with transforming growth factor beta 1 (TGF-β1) or high glucose. Several common target genes were identified for miRNAs within the megacluster, including trinucleotide repeat containing 6B (TNRC6B), CUGBP Elav-like family member 2 (CUGBP2), cytoplasmic polyadenylation element binding protein 4 (CPEB4), pumilio RNA binding family member 2 (PUM2), PHD finger protein 21A (BHC80), activating transcription factor 3 (ATF3), and ER degradation enhancing alpha-mannosidase like protein 3 (EDEM3). Expression of these transcripts was decreased in the glomeruli from diabetic mice compared with control animals, and in mouse mesangial cells treated with TGF-β1 or high glucose relative to control conditions. Treatment of mesangial cells with siRNA against Chop (the endoplasmic reticulum, stress-related transcription factor), which was identified as a regulator of lnc-MGC, resulted in reduced lncRNA expression under conditions of hyperglycemia and TGF- β1 treatment. Levels of lnc-MGC, as well as the candidate cluster miRNAs, were significantly decreased in glomeruli of diabetes-induced Chop-knockout mice. Treatment with a single oligonucleotide targeting lnc-MGC affected expression of the host lncRNA, the resident miRNA cluster, target genes of the cluster miRNAs, and profibrotic genes, and decreased characteristics of diabetic nephropathy in mice and human mesangial cells. This comprehensive study uncovered a wide range of functions for a new lncRNA and its component miRNAs in diabetic kidney disease, and introduced a novel target for potential pharmacological intervention.

Another study of STZ-induced diabetic mice identified renal upregulation of the metastasis-associated lung adenocarcinoma transcript 1 (MALAT1) lncRNA compared to age-and sex-matched control animals [60]. Increased MALAT1 levels corresponded with upregulation of pro-inflammatory genes, including serum amyloid A3 (SAA3), tumor necrosis factor (TNF), and interleukin 6 (IL-6), while MALAT1 downregulation was found to prevent generation of reactive oxygen species (ROS) in endothelial cells under hyperglycemic conditions [60]. In mice exposed to inspiratory hypoxia, MALAT1 expression was upregulated in renal proximal tubule cells [60,61]. Together, these studies suggest a role for MALAT1 in processes related to inflammation and hypoxia within the context of diabetes; additional work is necessary to further elucidate the role of this lncRNA in biological processes that underlie the development of renal dysfunction in T2D.

Expression levels of another lncRNA, MIAT (myocardial infarction associated transcript), were reduced in renal tubules of diabetic rats and renal tubular epithelial (HK-2) cells cultured under hyperglycemic conditions [63]. Functional characterization identified a relationship between MIAT and nuclear factor erythroid 2–related factor 2 (Nrf2), which regulates cellular resistance to oxidant exposure. Overexpression of MIAT reversed high glucose-mediated inhibition of Nrf2 and enhanced Nrf2 stability, which increased cell viability. Downregulation of MIAT corresponded with decreased cell viability, which was reversed by Nrf2 overexpression [63].

Our group identified plasmacytoma variant translocation 1 (PVT1) as a potential locus for diabetic end-stage renal disease using a genome-wide association approach in Native Americans [53], and PVT1 variants have since been associated with diabetic kidney disease in other populations [106,107]. In human mesangial cells, PVT1 expression was upregulated by glucose [69]. Downregulation of PVT1 in human mesangial cells corresponded with reduced mRNA and protein levels of fibronectin and collagen type IV α 1, and regulators of ECM proteins, TGF-β1 and plasminogen activator inhibitor [69], suggesting that this lncRNA may mediate the development and progression of diabetic kidney disease through mechanisms involving both increased synthesis and decreased degradation of specific ECM components.

Like lnc-MGC, PVT1 hosts a number of miRNAs within its primary sequence [108]. We found that four of the six annotated miRNAs mapping to this locus, miR-1205, miR-1207-3p, miR-1207-5p, and miR-1208, were upregulated in response to hyperglycemia in human mesangial cells [109], indicating that PVT1 may affect ECM accumulation through miRNA-mediated actions. Similar findings of host noncoding RNAs with resident miRNAs have been reported, suggesting a deeper complexity between lncRNAs and miRNAs in the regulation of transcriptional activity. For example, lncRNA RP23-298H6.1-001 hosts miR-216a and miR-217, all of which were induced by TGF-β1 in glomerular mesangial cells [110]. Both miRNAs targeted PTEN (phosphatase and tensin homologue), leading to glomerular mesangial cell survival and hypertrophy. Likewise, CJ241444 hosts miR-192, and expression levels of both increased in the presence of TGF-β1 [111].

### 1.5. LncRNAs Involved in Diabetic Retinopathy

Diabetic retinopathy is a microvascular complication of diabetes that affects approximately one-third of all patients with diabetes and is the most common cause of blindness in diabetic patients [112]. Poor glycemic control, genetic susceptibility, diabetes duration, hypertension, hyperlipidemia, and albuminuria are risk factors for the development and progression of diabetic retinopathy [113,114,115], although the pathogenesis of the disease is considered to be multifactorial. A number of studies have been performed to assess the role of lncRNAs in the development of diabetic retinopathy. To date, the majority of lncRNAs identified in these studies are ones that have been previously implicated in the development and progression of T2D and diabetic kidney disease (Table 1).

Expression profiling of retinal lncRNAs in STZ-induced diabetic mice identified over 300 dysregulated transcripts, including 214 downregulated and 89 upregulated lncRNAs [116]. The highest enrichment of biological processes targeted by lncRNA-co-expressed transcripts included cellular response to stress, integral to membrane, and structural molecule activity. KEGG pathway analysis demonstrated enrichment in axon guidance, mitogen-activated protein kinase (MAPK) signaling complement and coagulation cascades, chemokine signaling, and pyruvate metabolism. MALAT1 was significantly upregulated in retinas of STZ-induced diabetic rats and db/db mice [62], a retinal endothelial cell line (RF/6A cells), and aqueous humor samples and fibrovascular membranes from diabetic patients [116]. Downregulation of MALAT1 expression corresponded with improvements in markers of visual function (reduced amplitudes of a-, b-, and oscillatory potential waves) and retinal vessel function (reduced pericyte loss, capillary degeneration, microvascular leakage, and retinal inflammation) [62]. MALAT1 knockdown also alleviated retinal inflammation in vivo and reduced retinal endothelial cell proliferation, migration, and tube formation in vitro, through a p38 mitogen-activated protein kinase signaling mechanism [62]. These results, combined with investigations of MALAT1 in diabetic kidney disease, suggest that upregulation of this lncRNA represents a pathogenic mechanism for diabetes-induced microvascular dysfunction. In our opinion, inhibition of MALAT1 expression as a potential anti-angiogenic therapy for diabetes-related microvascular complications merits further exploration.

ANRIL, discussed earlier in the context of T2D, has also been explored in the regulation of vascular endothelial growth factor (VEGF), a key promoter of angiogenesis [117]. In human retinal endothelial cells (HRECs), expression of both VEGF and ANRIL was increased in the presence of 25 mM glucose compared to osmotic controls [50]. Further, knockdown of ANRIL expression in STZ-induced diabetic mice corresponded with attenuation of increased VEGF transcript and protein levels in retinal tissues compared to non-diabetic animals [50]. VEGF is regulated by miR-200b through the histone acetylator p300 [118] and the transcriptional-repressive polycomb repressive complex 2 (PRC2) complex [119]. In ANRIL knockout animals, levels of the PRC2 subunit, EZH2 (enhancer of zeste homolog 2), were significantly reduced, while siRNA-mediated ANRIL downregulation in HRECs reversed glucose-induced p300 upregulation and miR-200b downregulation [50]. In the presence of high glucose levels, ANRIL interacted directly with both p300 and EZH2. Together, these results show that ANRIL, in concert with p300, miR-200b, and EZH2, regulates VEGF expression and function in diabetic retinopathy.

Expression of MEG3, also discussed above, was decreased in STZ-induced diabetic mice and RF/6A endothelial cells exposed to hyperglycemia and hydrogen peroxide [67]. MEG3 knockdown in diabetic mice resulted in increased levels of acellular capillary formation, microvascular leakage, and inflammatory proteins. In RF/6A cells, MEG3 knockdown increased cell viability, proliferation, migration, and tube formation. In endothelial cells, PI3K/Akt signaling regulates angiogenesis, proliferation, and microvascular permeability [120]. MEG3 knockdown increased levels of phosphorylated PI3K and Akt, but without affecting total levels of either protein [67]. Involvement in the PI3K/Akt signaling pathway suggests that MEG3 may play a role in angiogenesis. Further investigation into this relationship may yield a better understanding of retinal vascularization and provide novel targets for therapeutic intervention.

Expression levels of MIAT, which is expressed in the retinal pigment epithelium layer, outer nuclear layer, inner nuclear layer, and ganglion cell layer in human and rat retinas, were increased in the presence of diabetes, as measured in STZ-induced diabetic rats, db/db mice, and fibrovascular membranes of individuals with diabetes, compared with the relevant controls [64]. In vitro, high glucose treatment of RF/6A cells increased MIAT expression in a time-dependent manner. MIAT knockdown in STZ-induced diabetic rats did not affect body weight or blood glucose levels, but corresponded with improvements in visual function and partial reversal of a-wave, b-wave, and oscillatory potentials. In these animals, MIAT downregulation also decreased the number of apoptotic retinal cells and attenuated retinal vessel impairment and retinal vascular leakage.

MIAT contains putative binding sites for four miRNAs, one of which, miR-150-5p, was found to directly target the lncRNA and regulate its expression in vitro [64]. Injection of miR-150-5p mimic or inhibitor into vitreous cavities of diabetic rats resulted in reduction or upregulation of *MIAT* levels, respectively. Of note, while hyperglycemia increased MIAT levels in diabetic rats, miR-150-5p elevated expression even further, suggesting that miR-150-5p regulates MIAT expression in vivo. Interestingly, miR-150-5p also targets *VEGF*, and co-transfection experiments with the 3′-untranslated region of *VEGF* cloned into a luciferase vector (RLuc-VEGF-WT), miR-150-5p mimic, and MIAT showed miRNA-mediated reductions in RLuc-VEGF-WT activity, which was partially alleviated by MIAT overexpression [64]. Overexpression of MIAT corresponded with elevated *VEGF* levels in RF/6A cells, which were reduced in the presence of increasing amounts of miR-150-5p. Conversely, miR-150-5p overexpression decreased *VEGF* expression, which was attenuated in the presence of increasing amounts of MIAT. Together, these results describe a potential mechanism by which crosstalk among MIAT, miR-150-5p, and VEGF might regulate endothelial cell function under hyperglycemic conditions.

The observation that overexpression of MIAT is associated with deterioration of retinal function in diabetes is in sharp contrast with results showing detrimental effects of reduced MIAT levels on cell viability in renal tubules [63]. One possible reason for the disparate findings between retina and renal tubule may stem from different roles played by MIAT in each specific cell type. Inhibition of MIAT expression under diabetic conditions improved retinal microvascular dysfunction in vivo, and decreased endothelial cell proliferation, migration, and tube formation in vitro by acting as a competing endogenous sponge for miR-150-5p, and creating a feedback loop with *VEGF* and miR-150-5p [64]. The mechanism by which MIAT affects renal tubule function within the context of diabetes remains to be determined; exploration of the MIAT-miR-150-5p relationship in renal tubule cells is also likely to shed light on these differences.

Retinal ganglion cell (RGC) injury is one of the important pathological features of diabetes-induced retinal neurodegeneration. Because of its role in central nervous system development and as a candidate for myopia, Sox2 overlapping transcript (SOX2OT) was investigated as a regulator of retinal neural function [121]. In RGCs exposed to high glucose or oxidative stress, SOX2OT levels were reduced in a time-dependent manner. Retinal SOX2OT levels were also lower in STZ-induced diabetic mice compared to untreated control animals. Amplitudes of a-wave, b-wave, and oscillatory potentials were significantly reduced in diabetic mice, but knockdown of *SOX2OT* expression ameliorated visual function and partially reversed the decreased trend of a-wave, b-wave, and oscillatory potentials. SOX2OT knockdown protected RGCs against high glucose-induced injury in vitro and affected diabetes-induced oxidative stress response retinas from diabetic mice and in RGC culture. NRF2 and heme oxygenase 1 (HO-1) are important factors in the regulation of oxidative stress [71]. Knockdown of *SOX2OT* expression increased NRF2 and HO-1 protein levels and disrupted interaction between NRF2 and Kelch-like ECH-associated protein 1 (KEAP1), which could interfere with targeting the complex for proteasomal degradation. In addition, *SOX2OT* knockdown contributed to NRF2 protein accumulation and nuclear translocation. Thus, *SOX2OT* knockdown may play a protective role in high glucose-mediated cell injury via interactions with NRF2 and HO-1.

### 1.6. LncRNAs as Targets for Therapeutic Intervention

Approaches used to target protein-coding gene expression can be applied for the manipulation of lncRNAs in vivo [122]. For example, lncRNA levels can be inhibited using RNA interference technology [123], which makes use of small RNA molecules that specifically bind to complementary sequences and inhibit expression of lncRNA targets. Many siRNA-based therapeutics are currently in clinical trials [124]. Because siRNA-based therapeutics have been shown to inhibit gene expression in cancer and other diseases, without significant toxicity in animal models [125], these molecules may be effective in targeting the expression and function of lncRNAs. Initial studies using MALAT1 siRNAs demonstrated inhibition of cell migration, growth, and invasion in prostate cancer cells [126], while siRNAs directed against HOTAIR have been shown to reduce matrix invasion in breast cancer cells [20].

Antisense oligonucleotides (ASO) and locked nucleic acid GapmeRs (Exiqon, Vedbaek, Denmark), also block lncRNA activity or induce enzyme-mediated degradation. For example, ASOs targeting MALAT1 in mouse lung cancer cells showed specific accumulation in tumor cells and associated stromal cells and reduced tumor metastasis [127]. GapmeRs directed against MALAT1 significantly inhibited blood flow recovery and capillary density, and impaired endothelial cell proliferation, leading to decreased vascular growth following hind limb ischemia in mice [128].

Despite the potential therapeutic value of siRNAs, ASOs, and GapmeRs in treating human disease, the effect of these pharmacological approaches may be complicated by the lengthy sequences and complex secondary structures of lncRNAs. Oftentimes, silencers are also unstable, interfere with nucleases, and have a low target affinity that necessitate use of higher concentrations or a cocktail of many silencers targeting more than one region of lncRNA, which could result in toxicity and off-target binding [124]. Thus, these challenges will need to be addressed before the use of lncRNA silencing can as therapeutic treatment strategies.

Blocking of molecular interactions can also be used to target lncRNAs. Small molecule inhibitors or complementary oligonucleotides that mask protein-binding sites on lncRNAs, and consequently interfere with lncRNA-protein interactions, can inhibit functional effects of these molecules. While the molecular mechanisms underlying lncRNA functions are not fully known, features such as molecular scaffolds for protein complexes and guides for chromatin complexes, may serve as targets for interference. For example, disruption of the interaction between HOTAIR with PRC2 may decrease metastasis in breast cancer [129].

LncRNAs can also be targeted through disruption of secondary structure. For example, small molecule inhibitors that bind to target lncRNAs can alter secondary structure characteristics. Similarly, complementary oligonucleotides targeting a specific sequence in the lncRNA can be used to interfere with native folding. In cases where loss of a protective lncRNA leads to the development of disease, gene therapy approaches may be used to restore levels of the missing molecule.

In parallel with their role in disease pathogenesis, lncRNAs may also serve as markers of disease status and aid in the diagnosis, prognosis, and clinical management of disease, particularly cancer [1,2]. As noted above, elevated hepatic levels of HULC in hepatocellular carcinoma were mirrored in peripheral blood [21] and plasma [35] of affected individuals. Similarly, urinary MALAT1 levels were explored to predict the risk of prostate cancer before biopsy in both discovery and multi-center validation phases [130]. The MALAT1 score was significantly higher in men with a positive biopsy compared to those with a negative one and a higher area under curve (AUC) was observed for the MALAT-1 score versus total prostate specific antigen (PSA) and percent free PSA in patients with PSA values of 4.0–10 ng/mL. The authors speculated that testing of urinary MALAT1 levels would prevent up to ~50% of unnecessary biopsies in patients with diagnostically meaningful PSA levels (i.e., 4–10 ng/mL), without missing any high-grade cancers.

Exploration of lncRNAs as biomarkers for pre-diabetes and T2D is already underway. In a profiling study of 84 lncRNAs using serum samples from five diabetic and non-diabetic individuals, levels of growth arrest specific 5 (GAS5) were significantly decreased in affected patients [131]. In an expanded analysis of *GAS5* levels in 96 serum samples, lncRNA expression was decreased in patients with HbA1c >5.9. Receiver operating characteristic (ROC) analysis was performed in these 96 samples to determine the optimal cutoff of GAS5 for diagnosis of T2D. The ROC analysis revealed an optimal cutoff GAS5 value of ≤10 ng/µL, and individuals with absolute GAS5 <10 ng/µL had significantly greater odds of having diabetes (odds ratio, OR = 11.79 (95% CI: 3.97, 37.26), *p* < 0.001) than those with higher GAS5 levels. The estimated AUC of ROC was 0.81 with 85.1% sensitivity and 67.3% specificity in distinguishing non-diabetic from diabetic individuals. The positive predictive value of GAS5 concentration was calculated to be 71.4%. While these results are promising, this study was limited to a relatively small cohort of Caucasian men recruited from a single site. Exploration of GAS5 in other populations with a greater representation of females and ethnic diversity will be necessary to better appreciate the potential of this lncRNA to serve as a biomarker for T2D.

A recent study profiled lncRNA expression in peripheral blood samples from six T2D patients and six unaffected individuals [132]. Seventeen lncRNAs were differentially expressed between the two groups, five of which were selected for validation in a second cohort comprised of 20 pre-diabetic individuals, 20 T2D patients, and 20 healthy controls. Of these, expression levels of three lncRNAs increased linearly from the control group to the pre-diabetes group to the T2D group. ENST00000550337.1 showed the highest AUC in ROC curve analysis and the lowest p-value, and was chosen for validation in a third cohort consisting of 63 pre-diabetic individuals, 64 T2D patients, and 60 healthy controls control group. In this cohort, levels of ENST00000550337.1 increased linearly in individuals from the control group to the pre-diabetes group to the T2D group, with a 2.2-fold change between the control and pre-diabetes groups and a 1.6-fold change between the pre-diabetes and T2D groups. The AUC, sensitivity and specificity of *ENST00000550337.1* in pre-diabetes and T2D were 0.714 ([0.624–0.804], *p* < 0.001), 0.81, and 0.533, and 0.701 ([0.609–0.793], *p* < 0.001), 0.672, and 0.698, respectively. Odds ratios adjusted for smoking, hypertension, BMI, total cholesterol, triglycerides, HDL cholesterol, and LDL cholesterol were 1.411 ([1.159–1.718], *p* = 0.001) and 1.269 ([1.106–1.455], *p* = 0.001) for pre-diabetes and T2D, respectively. Although this investigation was a single-site study conducted in Asian individuals, these results suggest that ENST00000550337.1 may be able to differentiate between pre-diabetes and T2D. Comparisons with other clinical diagnostic criteria and investigations in ethnically diverse cohorts are necessary to establish the utility of this lncRNA as a biomarker for impaired glucose metabolism.

## 2. Conclusions

For many years, dysregulated or dysfunctional changes in DNA, RNA, and proteins were considered the primary factors underlying the development of human disease, including diabetes. The discovery of dysregulated islet-specific lncRNAs adds a new layer of complexity to the molecular etiology of T2D and related microvascular complications of the kidney and eye. Although limited in number, the studies reported to date point to a role for lncRNAs in the regulation of processes related to T2D and diabetic complications, such as, β-cell identity and function. Functional characterization of islet-specific lncRNAs is underway [73], although a substantial amount of work is needed to understand the relative importance of these molecules in the pathogenesis of T2D. In addition, many of the functional lncRNAs identified in non-human models are limited by either the absence of that lncRNA in humans or the non-human homolog may have a different function than the human one. Also, lncRNAs appear to be poorly conserved among species, so that studies of mouse lncRNAs and their potential role in human T2D should be viewed with caution. The relative dearth of lncRNA annotation coupled with the lack of deep understanding of the complex array of functions performed by lncRNAs also limits the use of these molecules as therapeutic targets in clinical practice at this time. Further, the role of lncRNAs within the context of diabetic kidney disease and diabetic retinopathy is a relatively new area of exploration and more research studies of the mechanisms by which these transcripts alter molecular and cellular processes during disease pathogenesis are critical.

The findings summarized here, in combination with emerging results, are expected to yield new insights into the complex pathogenesis of T2D and related complications, and may eventually lead to the identification of novel cell-specific therapeutic targets with limited effects in other cell types, as well as improved methods for the T2D diagnosis and prediction of disease trajectory.

## Figures and Tables

**Table 1:** Long noncoding RNAs (lncRNAs) associated with type 2 diabetes (T2D) and related microvascular complications.

lncRNA	Name	Phen	Major Findings	Reference
ANRIL	antisense noncoding RNA	T2D	may affect β-cell mass	[49]
		DR	regulates VEGF expression in retina	[50]
βlinc1	β-cell long intergenic noncoding RNA	T2D	associated with β-cell loss	[51]
CYP4B1-PS1-001	cytochrome P450, family 4, subfamily b, polypeptide 1, pseudogene 1	DKD	may regulate proliferation and fibrosis in mesangial cells	[52]
E330013P06 (E33)		T2D	promotes macrophage inflammation	[53]
ENSMUST-00000147869		DKD	protects mesangial cells from proliferation and fibrosis	[54]
Gm4419	predicted gene 4419	DKD	regulates levels of pro-inflammatory cytokines and ECM genes	[55]
H19	imprinted maternally expressed transcript	T2D	associated with increased birth weight; higher expression in T2D patients	[56,57]
HI-LNC901		T2D	implicated in islet function	[58]
Lnc-MGC	lncRNA-megacluster	DKD	affects pro-fibrotic gene expression	[59]
MALAT1	metastasis-associated lung adenocarcinoma transcript 1	DKD	promotes inflammation and hypoxia within the context of diabetes	[60,61]
		DR	associated with markers of visual and retinal vessel function	[62]
MIAT	myocardial infarction associated transcript	DKD	regulates resistance to oxidant exposure	[63]
		DR	attenuates retinal vessel impairment and vascular leakage	[64]
MEG3	maternally expressed 3 gene	T2D	associated with impaired glucose tolerance, glycogen content, and insulin synthesis and secretion	[65,66]
		DR	modulates angiogenesis by PI3K/Akt	[67]
NEAT1	nuclear paraspeckle assembly transcript 1	T2D	regulates mTOR signaling pathway	[68]
PVT1	plasmacytoma variant translocation 1	DKD	regulates ECM components	[69]
PLUTO	PDX1 associated lncRNA, upregulator of transcription	T2D	regulates PDX1 expression	[70]
SOX2OT	Sox2 overlapping transcript	DR	mediates glucose-induced retinal injury	[71]

Phen: phenotype; DKD: diabetic kidney disease; DR: diabetic retinopathy; VEGF: vascular endothelial growth factor; ECM: extra cellular matrix; PI3K: phosphatidylinositide 3-kinase; Akt: protein kinase B; mTOR: mechanistic target of rapamycin; PDX1: pancreatic and duodenal homeobox 1; Sox2: SRY-box 2.
